# Tumor-Associated Extracellular Matrix Obstacles for CAR-T Cell Therapy: Approaches to Overcoming

**DOI:** 10.3390/curroncol32020079

**Published:** 2025-01-30

**Authors:** Ilya Klabukov, Alexander E. Kabakov, Anna Yakimova, Denis Baranovskii, Dmitry Sosin, Dmitry Atiakshin, Michael Ignatyuk, Elena Yatsenko, Victoria Rybachuk, Ekaterina Evstratova, Daria Eygel, Dmitry Kudlay, Vasiliy Stepanenko, Peter Shegay, Andrey D. Kaprin

**Affiliations:** 1 National Medical Research Radiological Centre of the Ministry of Health of the Russian Federation, Koroleva St. 4, 249036 Obninsk, Russia; 2 A. Tsyb Medical Radiological Research Center—Branch of the National Medical Research Radiological Centre of the Ministry of Health of the Russian Federation, Zhukova St. 10, 249036 Obninsk, Russia; 3 GMP-Laboratory for Advanced Therapy Medicinal Products, Patrice Lumumba Peoples’ Friendship University of Russia (RUDN University), Miklukho-Maklay St. 6, 117198 Moscow, Russia; 4 Obninsk Institute for Nuclear Power Engineering of the National Research Nuclear University MEPhI, Studgorodok 1, 249039 Obninsk, Russia; 5 University Hospital Basel, Basel University, 4001 Basel, Switzerland; 6 Centre for Strategic Planning and Management of Biomedical Health Risks of the Federal Medical Biological Agency, 119121 Moscow, Russia; 7 Scientific and Educational Resource Center for Innovative Technologies of Immunophenotyping, Digital Spatial Profiling and Ultrastructural Analysis, Patrice Lumumba Peoples’ Friendship University of Russia (RUDN University), 117198 Moscow, Russia; 8 Immunology Department, Institute of Immunology FMBA of Russia, 115552 Moscow, Russia; 9 Department of Pharmacognosy and Industrial Pharmacy, Faculty of Fundamental Medicine, Lomonosov Moscow State University, 119992 Moscow, Russia; 10 Institute of Pharmacy, Sechenov First Moscow State Medical University (Sechenov University), 119435 Moscow, Russia

**Keywords:** basement membrane, cancer, chimeric antigen receptor, CAR-T cell therapy, extracellular matrix, fibrosis, gene engineering, lymphocyte, oncomatrix, solid tumors, tumor microenvironment

## Abstract

Chimeric antigen receptor (CAR)-T cell therapy yields good results in the treatment of various hematologic malignancies. However, the efficacy of CAR-T cell therapy against solid tumors has proven to be limited, primarily because the tumor-associated extracellular matrix (ECM) creates an intractable barrier for the cytotoxic CAR-T cells that are supposed to kill cancer cells. This review unravels the multifaceted role of the tumor-associated ECM in impeding CAR-T cell infiltration, survival, and functions within solid tumors. We analyze the situations when intratumoral ECM limits the efficacy of CAR-T cell therapy by being a purely physical barrier that complicates lymphocyte penetration/migration and also acts as an immunosuppressive factor that impairs the antitumor activities of CAR-T cells. In addition, we highlight promising approaches such as engineering CAR-T cells with improved capabilities to penetrate and migrate into/through the intratumoral ECM, combination therapies aimed at attenuating the high density and immunosuppressive potential of the intratumoral ECM, and others that enable overcoming ECM-related obstacles. A detailed overview of the data of relevant studies not only helps to better understand the interactions between CAR-T cells and the intratumoral ECM but also outlines potential ways to more effectively use CAR-T cell therapy against solid tumors.

## 1. Introduction

Cancer is one of the leading causes of death worldwide, claiming millions of lives each year. An estimated 20 million people will be diagnosed in 2022, and the number is expected to increase by 77% to 35 million by 2050 [1]. Despite the significant advances in early diagnosis and therapy of tumors, many malignancies remain difficult to cure, underscoring the need for further development of the effective methods of cancer treatment and prevention [2].

**Figure 1:**
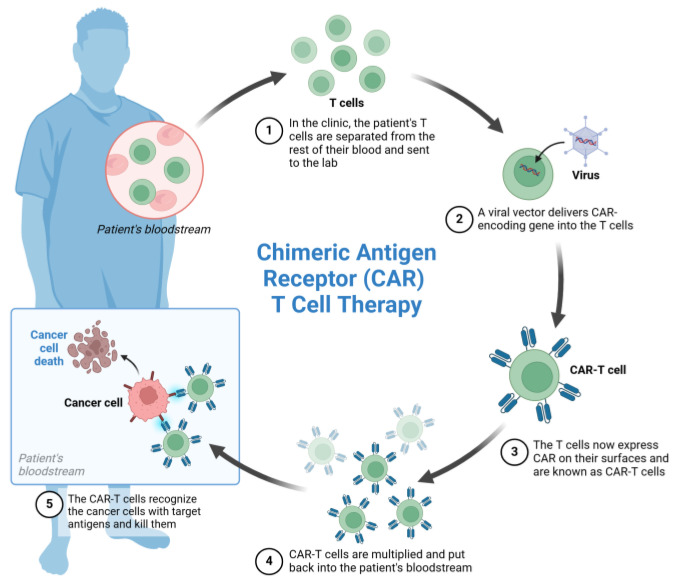
Schematic illustration of basic principle of CAR-T cell therapy: 5-stage circuit including isolation of patient’s T-lymphocytes (1), infection of them with viral vector with gene-encoding CARs (2), identification of CAR-expressing T cells (3), propagation of CAR-T cells and introducing of them into patient’s body (4), and CAR-T cell-induced killing of cancer cells within patient’s body (5). Created with Biorender.com.

Cancer treatments currently demonstrate remarkable advances with the advent of chimeric antigen receptor T-cell (CAR-T) therapy, a form of immunotherapy when patient’s T-lymphocytes are genetically modified to better recognize and attack malignant cells [3]. Briefly, fractions of T-cells harvested from a patient with cancer are infected with an artificially constructed viral vector to express specific fusion proteins that will enable the infected T cells to assemble antigen receptors of a new type, namely chimeric antigen receptors (CARs) with the programmed alterations in their extracellular and intracellular domains. Thanks to those CARs, the T-lymphocytes acquire the following novel, critically important activities: (i) CAR-T cells become able to target a specific molecule ligand (e.g., CD19) exposed on the surface of cancer cells, and (ii) CAR-T cells aim to kill target cancer cells after their CARs bind to the corresponding ligands on the surface of cancer cells. Being multiplied and injected into a patient’s organism, such ex vivo engineered cytotoxic T-lymphocytes begin to attack and eliminate cancer cells (Figure 1), resulting in a therapeutic effect.

CAR-T cell therapy already showed its efficiency on the treatment of hematological malignancies (leukemias, lymphomas, multiple myeloma) compared with chemotherapy [4,5]. Despite the acknowledged success of CAR-T cell therapy, particularly in the treatment of certain blood cancers, the efficacy of CAR-T cell therapy in solid tumors is limited [4,5]. There are several reasons why this therapeutic modality is ineffective toward solid tumors, but the main reasons appear to be specific features of the tumor microenvironment (TME) and, particularly, the tumor-associated extracellular matrix (ECM) [6,7].

In recent years, several review papers were published in which the intratumoral ECM was considered not as a static scaffold surrounding cancer cells and stromal cells, but as a complex, multicomponent structure that actively contributes to tumor progression and resistance to therapies, including CAR-T cell therapy [8,9,10,11]. Some authors discussed ECM-involving mechanisms that impair T cell infiltration into tumors and repress the T cell viability/functions, thus helping malignant cells to evade T cell attacks [10]. While research publications about various attempts to overcome ECM-related obstacles to CAR-T cell therapy are piling up, Nguyen et al. [11] tried to summarize in their review some promising strategies such as engineering CAR-T cells with improved infiltration capabilities and using combination therapies simultaneously targeting tumor cells and intratumoral ECM. However, to date, no significant reviews have been published that include both a detailed description of the ECM-associated factors hindering CAR-T cell therapy and a comparative analysis of all potential approaches to overcome this problem.

That is why we present here a complete, comprehensive review by first linking CAR-T cell therapy failures in solid tumors to specific peculiarities in the composition, structure, and properties of the intratumoral ECM, and, second, analyzing numerous approaches and strategies aimed at overcoming or minimizing the barriers posed by the intratumoral ECM for CAR-T cells. This review calls for further careful studies of the role of ECM in hindering CAR-T cell therapy in solid tumors and for the development of innovative technologies that would make intratumoral ECM more permeable to CAR-T cells.

## 2. ECM as Major Component of Tumor Stroma: Composition/Structure, Properties, and Contribution to Pathogenesis of Cancer

### 2.1. Composition, Structure, and Properties of Intratumoral ECM

**Figure 2:**
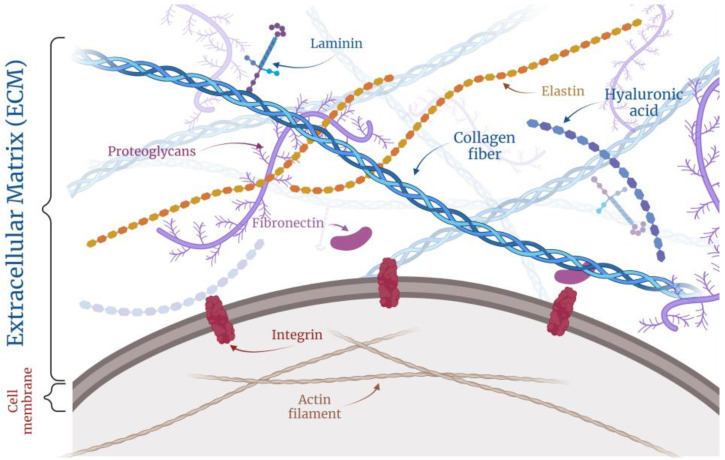
Schematic illustration showing major components of ECM and its interactions with cell surface. Note: Multicomponent composition of ECM includes glycoproteins, proteoglycans, and polysaccharides which can form fibrillar structures and interact with transmembrane integrins anchored to the actin cytoskeleton. Created with Biorender.com.

The extracellular matrix (ECM) is a central component of the tumor stroma and plays a critical role in cancer pathogenesis due to its intricate composition, structure, and properties [12]. Consisting of a complex network of (glyco)proteins, such as collagen, elastin, fibronectin, laminin, and others, and also proteoglycans and glycosaminoglycans, ECM provides structural support to tissues and mediates critical biochemical and biomechanical signals that regulate cell phenotype and cell behavior [13], as seen in Figure 2.

Being a supporting framework for cells within tissues and organs, the ECM also functions as a mediator–conductor for the exchange of ions and molecules that constantly occurs between the living cell and its microenvironment. In order to reach the cell, water, ions, O_2_, nutrients, growth factors, hormones, drugs, etc. must diffuse through the thickness of the ECM. In turn, CO_2_ and all other molecules coming out of cells, as well as secreted endosomes, first pass through the ECM layer.

In the context of cancer, the intratumoral ECM undergoes significant remodeling that is characterized by changes in its composition and covalent cross-linking of its components, resulting in increased stiffness and altered mechanical properties [14,15]. This remodeling not only facilitates tumor progression by promoting cancer cell proliferation, invasion, and metastasis, but also contributes to the creation of an immunosuppressive microenvironment that impedes effective antitumor immune responses. In addition, the altered ECM can sequester growth factors and cytokines, modulating their availability and activity, thereby influencing cancer cell survival, angiogenesis, and resistance to therapy [16]. The dynamic interplay between cancer cells and ECM underscores its importance in the pathogenesis of cancer and highlights ECM not only as a static, passive scaffold, but as a critical player in the TME that actively contributes to cancer progression and represents a potential target for therapeutic intervention [17,18].

Fibrillar structures within the ECM, composed primarily of collagen, elastin, and fibronectin, provide tensile strength and elasticity to tissues. Collagen fibers, the most abundant protein constituent in ECM, form a triple helix structure that assembles into fibrils and then into larger fibers, creating a scaffold that fills the intercellular spaces and supports tissue integrity. Elastin fibers, on the other hand, provide elasticity, allowing tissues to return to their original size/shape after stretching or contracting. Fibronectin is a glycoprotein that facilitates cell adhesion and migration by attaching cells to the collagen matrix [19].

Laminin belongs to a family of glycoproteins with a heterotrimeric structure comprising three different chains (α, β, and γ). The members of this family are contained in the ECM and influence cell adhesion, migration, and differentiation. Importantly, laminin is the obligatory constituent of the basement membrane (or basal lamina), a thin monolayer of the ECM on which cells of the epithelium and endothelium sit. Another glycoprotein of the ECM, vitronectin, is also a cell adhesion factor that promotes tumor cell migration. All these major proteins are organized in a highly ordered fashion and define the mechanical properties and structural framework of the ECM. Collagen, laminin, fibronectin, and vitronectin interact with the extracellular domain of integrins (a family of transmembrane heterodimeric proteins anchored on actin filaments) and these site-specific interactions can trigger certain signaling pathways influencing cell shape/motility, metabolism, proliferation, and secretory activity [20].

Not so abundant but important components of ECM are tenascin and thrombospondin; these multifunctional matricellular proteins contribute to the regulation of cell–ECM interaction dynamics. Tenascin is represented by its several isoforms where the most studied one is tenascin-C, which is able to modulate cell adhesion. Thrombospondins are a subfamily of matricellular proteins that also play multiple roles in the ECM and can suppress tumor growth by inhibiting angiogenesis. Their roles are context-dependent and can exert either promoting or inhibiting effects on the processes of cell adhesion/migration, angiogenesis, and cancer progression [20].

Besides the above glycoproteins, the ECM usually contains large amounts of proteoglycans and glycosaminoglycans (GAGs). Proteoglycans are proteins that are highly glycosylated with GAGs, which are long, linear carbohydrate polymers. Both proteoglycans and GAGs play critical roles in maintaining the structural integrity of tissues, mediating cell signaling, and regulating the movement of various molecules through the ECM. GAGs, such as hyaluronic acid, chondroitin sulfate, heparan sulfate, and others, contribute to the hydration and resilience of the ECM due to their highly negative charge, which attracts water and cations [21]. The interplay between the ECM-associated proteoglycans, GAGs, and the surrounding ion/molecule pool creates a hydrated gel-like structure that locally mediates the water/ion balance and O_2_/CO_2_ turnover in the cellular microenvironment.

The stiffness of the ECM is determined by several factors, including the composition and density of its components, the degree of cross-linking between protein fibers, and the presence of specific ECM-modifying enzymes. The interprotein cross-linking as a result of the enzymatic activities of lysyl oxidases, lysine hydroxylases, and transglutaminases forms covalent bonds between collagen molecules, thereby increasing the tensile strength and stiffness of the ECM; analogous cross-linking, when it progresses and becomes excessive, leads to fibrosis (desmoplasia) [22]. The balance between ECM synthesis and degradation, regulated by matrix metalloproteinases (MMPs) and their inhibitors, also influences ECM stiffness. The mechanical properties of ECM are critical for cell differentiation, migration, and proliferation, and play an important role in tissue development/repair, and tumor progression [20,23].

It is generally accepted that the stiffness of intratumoral ECM may contribute to the pathogenesis of cancer. In vivo, the dense and fairly thick ECM surrounding tumor cells protects them by making it difficult for immune cells, antitumor antibodies, or drugs to contact their targets. Moreover, the stiffness of the intratumoral ECM slows down the diffusion of O_2_ and its entry into tumor cells, which is one of the reasons for the development of hypoxic stress and acidosis in the TME. Hypoxia in tumors is known to confer on them radioresistance, which is partly due to the so-called “oxygen effect”. In addition, hypoxia-stressed tumor cells reprogram their metabolism and signaling to adapt to hypoxic conditions, so that the newly formed cancer cell phenotypes become more resistant to chemotherapy and radiation therapy [24,25]. Both the intratumoral hypoxia [24,26] and the intratumoral ECM stiffness [27] are the factors stimulating the epithelial-to-mesenchymal transition (EMT) in cancer cells, which leads to the emergence of highly mobile malignant cells with a mesenchymal phenotype and some traits of cancer stem cells (CSCs). Such EMT-transformed (CSC-like) tumor cells pose a serious challenge for cancer treatment because they are capable of forming distant metastases and are resistant to many anticancer agents. Taken together, these facts delineate how intratumoral ECM stiffness directly and indirectly contributes to the pathogenesis of cancer.

### 2.2. Which Cells Produce and Modify Intratumoral ECM and What Are Their Stimuli/Drivers?

**Figure 3:**
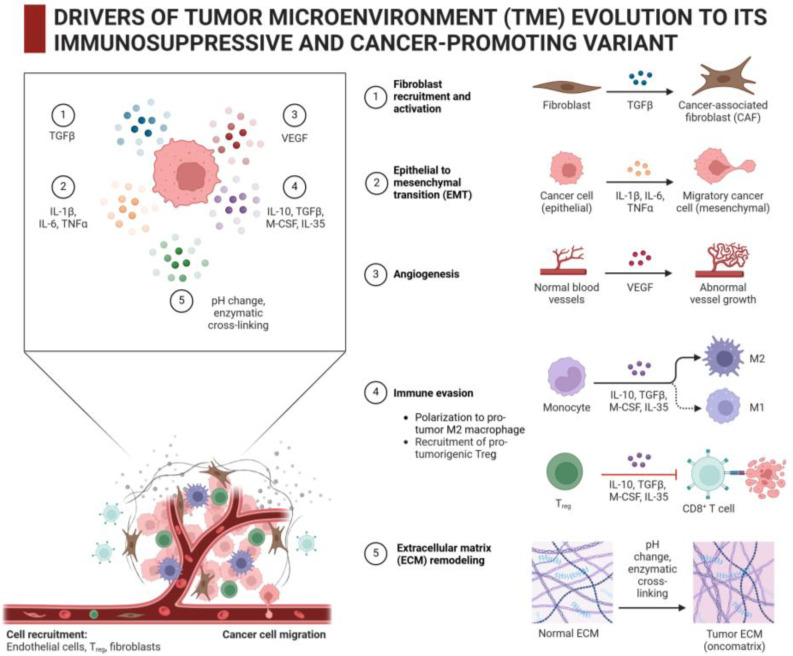
TME-associated factors and local processes contributing to ECM remodeling and immunosuppressive effects. Note how distinct microenvironmental factors, including lower pH, activities of extracellular enzymes, growth factors and cytokines, etc., mediate appearance of CAFs (1), epithelial-to-mesenchymal transition (EMT) in cancer cells (2), angiogenesis with development of aberrant vasculature (3), appearance of pro-tumor M2 macrophages and Treg cells, which both confer cancer cell resistance to attacks of cytotoxic T-lymphocytes (4), cross-linking and firming of intratumoral ECM (5). Created with Biorender.com.

Among various types of cells that participate in producing and modifying the intratumoral ECM, there are cancer-associated fibroblasts (CAFs), vascular endothelial cells and pericytes, immune cells, and malignant cells themselves [23,28]. The dynamic interaction between these cells and ECM plays a crucial role in TME remodeling that promotes tumor progression, metastasis spread, and tumor resistance to therapy [7,29], as seen in Figure 3.

CAFs are the most prominent stromal cells that are directly involved in the production and modification of intratumoral ECM. CAFs originate from various cellular precursors, including resident or tumor-recruited fibroblasts, mesenchymal stem cells, and endothelial cells [30]. CAFs actively generate ECM components such as collagen, fibronectin, hyaluronic acid, and others; in addition, these cells are also able to modify the surrounding ECM through the secretion of matrix metalloproteinases (MMPs), lysyl oxidases (LOXs), transglutaminases, and other enzymes [31]. It is transforming growth factor β (TGFβ) that is the master regulator of mechanisms of recruiting and transformation of normal fibroblasts into CAFs within the TME; this growth factor is produced by cancer cells and also by pro-tumor stromal cells such as Treg cells and M2 macrophages (Figure 3 and references [30,31]). TGFβ is known to be an attractant and mitogen for fibroblasts that stimulates their migration/proliferation into/inside the tumor stroma with the subsequent development of the pro-tumor CAF phenotype. Moreover, the interaction of TGFβ with its receptors in CAFs triggers certain signaling pathways that yield the enhanced synthesis/secretion of collagen and other ECM components [30,31]. The overstimulation of CAFs by excess TGFβ leads to tumor fibrosis [32]. Fibroblast growth factor (FGF), also abundantly generated by cancer cells and stromal macrophages, is another TME-associated mitogen that causes CAFs to proliferate [33]. Thus, TGFβ and FGF are molecular drivers of the accumulation of CAFs within the tumor stroma and the ECM overproduction by CAFs. These two parallel effects mediated by TGFβ and FGF, namely the increased ECM production and increased numbers of ECM producers (CAFs), are responsible for the formation of ECM-rich tumor stroma with increased mechanical stiffness, which is characteristic of fibrotic tumors.

Endothelial cells of the tumor vasculature can produce laminin, fibronectin, and other components of the basement membrane that is formed under the endothelium; additionally, some endothelium-derived factors can affect the adjacent ECM and neighboring cells. In vivo, along with the endothelial cell monolayer, the subendothelial basement membrane acts as a conditionally permeable barrier between the blood/lymph and any surrounding tissues [34]. Oxygen, nutrients, humoral and growth factors, and leukocytes including monocytes and T-cells—anything that comes from the bloodstream—must cross the subendothelial basement membrane of tumor vasculature to enter the tumor parenchyma. Aggressive solid tumors, usually with hypoxic regions, stimulate the intratumoral neovascularization, mainly due to the secretion of vascular endothelial growth factor (VEGF) by cancer cells and tumor-associated macrophages. Besides VEGF, FGF produced by cancer cells and macrophages is also a potent mitogen for vascular endothelial cells [35]. While tumor-stimulated (VEGF- and FGF-mediated) angiogenesis is one of the hallmarks of cancer, the abnormally rapid, unregulated development of the tumor vasculature often results in its structural and functional defects, including those in the subendothelial basement membrane. Consequently, the conditional permeability of the basement membrane for leukocytes may also be somewhat compromised in the abnormal tumor vasculature and this may complicate entry of T cells into the tumor [36,37].

It seems likely that pericytes, which are associated with the basement membrane and endothelial cells in capillaries and microvessels, produce/influence the surrounding ECM as well because of their role in stabilizing blood vessels and regulating blood flow [38].

The implication of monocyte-derived macrophages in TME remodeling is established: stromal macrophages may undergo pro-tumor M2 polarization and then contribute to tumor progression and metastasis by generating growth factors such as TGFβ, VEGF, EGF, FGF, and PDGF; proinflammatory factors such as TNFα, IL1β, IL6, and IL12; and immunosuppressive factors such as IL10 and PGE2 [39,40]. Although the tumor-associated macrophages by themselves are not the active producers of ECM components, these stromal cells secrete TGFβ and FGF and hence participate in the TGFβ- and FGF-mediated recruitment, proliferation, and functional stimulation of CAFs being the ECM-producers; this is the indirect involvement of stromal macrophages in the overproduction of intratumoral ECM. In addition, the tumor-associated macrophages affect the surrounding ECM by expressing ECM-modifying enzymes (MMPs, hyaluronidase, transglutaminase, and others). Some products of partial ECM degradation by stromal macrophages (e.g., fragments of GAGs) may also exert the immunosuppressive and cancer-promoting effects [41].

In addition to monocytes/macrophages, other immune cells must also contact and interact with the ECM to penetrate from the blood/lymph into tissues, including tumor tissue. For example, to overcome the subendothelial basement membrane barrier and subsequently move through adjacent layers of the ECM, T cells employ specific interactions between their integrins and laminin of the basement membrane [42], and also express heparanase, an enzyme that cleaves proteoglycans along the T cell migration path [43].

Last but not least, malignant cells in solid tumors themselves are the significant producers of both ECM components and ECM-modifying enzymes, including MMPs, LOXs, transglutaminase, heparanase, hyaluronidase, and others. Such ECM-related activities of tumor cells enable them to invade nearby tissues, metastasize, and resist the immune system and therapeutics [44,45]. As in the above situation with CAFs, TGFβ plays a key role in enhanced ECM production by cancer cells. By secreting TGFβ and concomitantly expressing its receptors, cancer cells stimulate themselves when the secreted TGFβ binds to its receptors on the cancer cell surface; this binding triggers specific signaling that leads to multiple cellular responses including the activation of synthesis of collagen and other ECM components [46]. In addition to this autostimulation of cancer cells by “their own” TGFβ, TGFβ produced by pro-tumor Treg cells and M2 macrophages can also stimulate cancer cells (together with CAFs) to overproduce the ECM.

Heat shock proteins (HSPs), particularly HSP47 and HSP90, appear to be implicated in ECM formation/remodeling by tumor cells. HSP47 (SERPINH1) is a molecular chaperone that localizes to the endoplasmic reticulum lumen and assists with the correct folding of procollagen and subsequent transport of collagen molecules; therefore, HSP47 is essential for the collagen synthesis by the cell. Suggestions are put forward about the roles of HSP47 as a fibrosis-promoting and cancer-promoting factor that can be therapeutically targeted [47,48]. Most likely the high level of HSP47 expression in cancer cells and CAFs contributes to collagen overproduction by them and the development of tumor fibrosis. In turn, HSP90 is one of the key chaperones and regulators of intracellular signaling [49]; in addition, some HSP90 molecules are transported by the cell into the extracellular space (the so-called extracellular HSP90 or eHSP90). There are many data indicating that eHSP90 secreted by cancer cells is actively involved in the remodeling of intratumoral ECM [50]. Specifically, eHSP90 is conducive to increased ECM deposition in TME by inhibiting the reinternalization of secreted fibronectin and catalyzing the assembly of thicker fibers composed of fibronectin and collagen. Furthermore, eHSP90 can modulate the stability/activities of ECM-modifying enzymes such as proteases (including MMPs), lysyl oxidases (including LOXL2), and others. It is eHSP90 that directly binds to collagen-1 and mediates the involvement of LOXL2 in the cross-linking and the alignment of thick collagen fibers in breast malignancies [50,51].

Such an enzyme as focal adhesion kinase (FAK), expressed in cancer cells, seems to be somehow implicated in the ECM overproduction-related tumor fibrosis: FAK hyperactivation revealed in human pancreatic cancer was shown to correlate with a formation of the fibrotic TME [52].

Thus, the cancer-promoting deposition of thick and dense ECM within the TME is a complex, multifactor process involving both malignant cells and stromal cells.

## 3. Intratumoral ECM as an Obstacle for CAR-T Therapy

The ECM built by cancer cells and stromal cells is one of the major factors responsible for the failure of CAR-T therapy toward solid malignancies. Such harmful effects of the tumor-associated ECM are mainly due to (i) its immunosuppressive potential and (ii) its augmented mechanical density (stiffness) that hinders T-lymphocyte infiltration and functioning; both of these points are considered in the present section.

### 3.1. Immunosuppressive Properties of Intratumoral ECM That Can Cause CAR-T Cell Exhaustion/Dysfunction

The altered ECM within solid tumors is a cancer-promoting factor that contributes to not only tumor progression, invasion, and metastasis spread, but also to the creation of an immunosuppressive microenvironment impeding effective antitumor immune responses. In contrast to such “short-living” and shortly acting immunosuppressors as TGFβ, IL10, IL35, PGE2, and others produced by cancer and stromal cells, in a context-dependent fashion, intratumoral ECM is the invariably presenting and constantly acting immunosuppressive factor in TME [53,54]. The immunosuppressive properties of the intratumoral ECM are manifested toward both the host immunity and tools of antitumor immunotherapy including antibody-based or antibody-conjugated agents and CAR-T cells.

**Figure 4:**
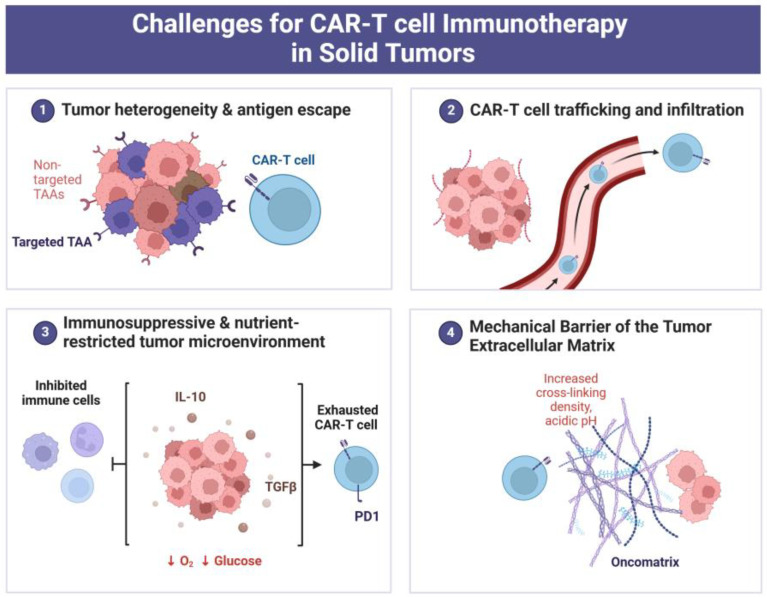
The four major challenges for applying CAR-T cell therapy against solid tumors. It is clearly seen in the figure that the efficacy of CAR-T cell therapy can be limited when tumor-directed CAR-T cells do not find their target antigens through masking their epitopes and/or propagation of cancer cells free of the target antigens (1); when CAR-T cells have difficulties with penetrating across the vascular wall into the tumor parenchyma and with their migration toward target cancer cells (2); when CAR-T cells undergo energy starvation, immunosuppression resulting from cancer cells, and exhaustion (3), and when mechanical properties of the ECM provide a physiological barrier for CAR T cells (4). It follows from the accompanying text of the present review that the intratumoral ECM is directly or indirectly implicated in all four challenges. Created with Biorencer.com.

Masking epitopes and steric inaccessibility to immune cell/antibody targeting tumor-associated antigens (i.e., “antigen escape”) are thought to be one of the major mechanisms by which constituents of the intratumoral ECM can help solid tumors evade immune surveillance [55,56]. The altered ECM in solid tumors represents an immunosuppressive barrier for cytotoxic T-lymphocytes. Similarly, the tumor-modified ECM impedes the infiltration and cytotoxic action of CAR-T cells engineered to recognize and kill cancer cells (Figure 4.). It occurs because the intratumoral ECM is able to sequester growth factors, cytokines, and chemokines that are required to maintain the viability, traffic, and cancer cell-killing capability of T-lymphocytes.

While the development of CAR-T cell exhaustion is known to be one of the major limitations in the efficacy of antitumor CAR-T cell therapy (Figure 4 and reference [57]), the immunosuppressive potential of intratumoral ECM appears to contribute to this phenomenon. For instance, the interactions between intratumoral ECM components and surface receptors on stromal cells belonging to the immune system can trigger signaling pathways that will lead to the exhaustion/dysfunction of T cells within the target tumor, thus reducing the beneficial effects of CAR-T therapy [58,59]. By another way, cancer cells can interact with the surrounding ECM, so that consequences of those interactions somehow affect CAR-T cells, causing their exhaustion. So, Zhang et al., using a model of CAR-T cell therapy with gastric cancer cells cultured in 3D collagen gel, found that collagen promotes CAR-T cell exhaustion through the activation of IL4I1-AHR signaling [60]. The revealed mechanism was initiated by integrin signaling that was triggered by the integrin αvβ1—3D collagen interaction and then realized via increased IL4I1 expression when one of the IL4I1 metabolites, namely kynurenic acid (KynA), promoted CAR-T cell exhaustion by stimulating the AHR pathway [60]. This ECM-related mechanism, if it takes place in vivo, may explain the resistance of gastric cancer to CAR-T cell therapy and implies a possibility to improve the therapeutic effect by neutralizing KynA or by inhibiting the AHR pathway.

The other ECM-associated immunosuppressive factor seems to be cancer-stimulated angiogenesis, resulting in abnormal development and functioning of tumor vasculature that can interfere with T-lymphocyte penetration from blood/lymphatic vessels into the tumor parenchyma (Figure 3 and Figure 4). The permeability of the vascular endothelium and adjacent basement membrane for T cells can matter herein [38,61,62,63]. In particular, the lack of laminin-α4 (one of the protein components of the ECM) in the subendothelial basement membrane of the aberrant vasculature in tumors renders this barrier less permeable for transmigrating T cells that leads to the attenuation of T cell infiltration through the vascular wall and capillaries [64]. If so, the altered (laminin-α4-deficient) subendothelial basement membrane of the tumor vasculature can impede CAR-T therapy of solid tumors.

The immunosuppressive potential is really manifested by various components of the tumor-associated ECM. It was found in an in vitro model that fibronectin, one of the major protein components of the ECM and a factor of cell adhesion, impairs the cytotoxic action of T-lymphocytes toward cancer cells adherent to a fibronectin-coated substrate [65]. If analogous phenomenon takes place in vivo, the fibronectin-enriched intratumoral ECM may decrease, by the same mechanism, the efficacy of CAR-T therapy toward solid tumors.

In turn, collagen, the most abundant and typical protein of EMC, can also function as an immunomodulator, while the excessive collagen accumulation in the tumor stroma is known to exert immunosuppressive (pro-tumoral) effects [55]. In vitro, collagen was demonstrated to inhibit the killing of cultured cancer cells by cytotoxic T cells [66]. Collagen of the intratumoral ECM directly interacts with T cells and also stimulates the transition of tumor-associated macrophages to their pro-tumor M2 phenotype with proinflammatory and immunosuppressive activities, to which the malignancy becomes able to withstand both host antitumor immune response and T cell immunotherapy [67].

Proteoglycans, the other dominant components of the ECM, can modulate the secretion and activity of cytokines, chemokines, growth factors, and adhesion molecules, and also cell receptor–ligand interactions and signaling. Heparan sulfate proteoglycans and chondroitin sulfate proteoglycans of the intratumoral ECM appear to be involved in pro-tumor responses that include the inhibition of T-lymphocyte attacks on cancer cells [55,68,69].

The abundant GAG component of the intrartumoral ECM is hyaluronic acid or hyaluronan. The abnormally high levels of biosynthesis and degradation of hyaluronic acid in the tumor stroma contribute to the crosstalk between coming immune cells and a growing solid malignancy [55]. Hyaluronidases expressed by both stromal cells and cancer cells split nearby hyaluronan, thus producing its smaller fragments, which can affect the phenotypic state and behavior of immune cells within the tumor stroma. In particular, the hyaluronan-enriched intratumoral ECM promotes the transition of tumor-recruited hyaluronidase 2-expressing myeloid-derived suppressor cells (MDSCs) of bone marrow origin to the tumor-associated PD-L1+ macrophages that actively remodel the TME toward its immunosuppressive and proinflammatory variant [70].

Some minor protein components of the intratumoral ECM, such as tenascin C, thrombospondin-1, and others, can also contribute to repressing both the antitumor immune surveillance and the cancer cell-killing function of introduced CAR-T lymphocytes [71,72].

Therefore, the immunosuppressive features of the intratumoral ECM represent a significant obstacle for CAR-T cell therapy, highlighting the need for innovative strategies to modify certain ECM-influencing factors of the TME and/or enhance CAR-T cell functionality in order to overcome such obstacles. Meanwhile, some of the ECM-associated immunosuppressive factors present in solid tumors seem surmountable and a number of approaches to solving this challenge are suggested.

### 3.2. ECM Stiffness as Factor That Interferes with T-Lymphocyte Infiltration into Tumor

It is generally accepted that the stiffness of tumor-associated ECM contributes to cancer pathogenesis. Understanding the mechanisms by which ECM stiffness inhibits T-lymphocyte targeting/killing of cancer cells is critical for developing new strategies aimed at increasing the efficacy of CAR-T therapy toward solid tumors. Importantly, the ECM components, such as collagen, fibronectin, proteoglycans, hyaluronan, and others, being overproduced by cancer cells and stromal cells, are involved in cross-linking and form a dense and rigid physical barrier that impedes CAR-T lymphocyte infiltration into the target malignancy [73,74].

The ECM stiffness-induced inhibition of T-lymphocyte infiltration into tumor tissue along with the impaired immunogenic death of cancer cells was established in many models with solid tumors of different tissue origin and localization [63,73,74,75,76]. In the case of osteosarcoma, this problem can yet be aggravated by a mineralization of the intratumoral ECM that additionally enhances the latter’s density and “impassability” for T-cells [75].

Apart from being a purely mechanical obstacle for T-cell traffic, the fibrotic stiffness of tumor-associated ECM can also impair the intratumor penetration/distribution of antibodies and immunoregulatory molecules (including cytokines and chemokines) that are necessary for the migration and functioning of cytotoxic T-lymphocytes [58,77,78]. In addition, the intratumoral ECM stiffness appears to promote CAR-T cell exhaustion that is one of the major causes of low efficacy of CAR-T cell therapy against solid tumors (Figure 4 and references [57,79]). When the cancer-altered architecture and high density of intratumoral ECM transmit biomechanical stress to entering (tumor-reactive) T-lymphocytes, this activates the transcription factor Osr2, which mediates cellular transcriptional response to biomechanical stress, finally resulting in the exhaustion of CD8+ T-cells or CAR-T cells [80]. At the molecular level, Osr2 recruits histone deacetylase 3 (HDAC3) to render epigenetic reprograming that turns off the expression of cytotoxic genes and is conducive to CD8+ T cell or CAR-T cell exhaustion [80].

Moreover, the extremely high density of tumor-associated ECM delays the diffusion of oxygen, glucose, and other nutrients in it, thus creating a hypoxic and nutrient-poor microenvironment; such stressful, energy-depriving conditions complicate the survival and therapeutic action of CAR-T cells within the solid tumor (Figure 4 and ref. [81]). As protein biosynthesis, enzyme secretion, and cell motility require energy (i.e., access to oxygen and metabolic substrates for ATP generation are required), CAR-T cells, being under conditions of energy starvation, will not be able to actively migrate through the dense intratumoral ECM. Taken together, the above ECM stiffness-associated factors worsen the outcome of CAR-T cell therapy of solid tumors with ECM-enriched fibrotic stroma.

Notably, while CAR-T cell therapy is thought to be rather successful toward hematological (non-solid) malignancies, ECM stiffness may matter there as well: the high levels of bone marrow fibrosis under B-acute lymphoblastic leukemia were shown to correlate with the lower levels of CD3+ T-cell infiltration in bone marrow and lower overall survival after CD19-directed CAR-T cell therapy [82].

Some researchers proposed to exploit in vitro test-systems with cancer cells cultured in 3D dense collagen or ECM matrices in order to evaluate the therapeutic potential of cytotoxic T-lymphocytes prior to clinical use of them in antitumor immunotherapy [60,83,84,85]. However, there are more suggestions for how to soften the stromal ECM and facilitate the CAR-T cell infiltration into solid tumors.

## 4. How to Overcome ECM-Associated Barriers in CAR-T Therapy?

The dense ECM, surrounding tumor cells, acts as a formidable and immunosuppressive barrier that hinders the penetration and therapeutic function of CAR-T cells within solid tumors. Obviously, to successfully apply CAR-T therapy against solid tumors, the above ECM-associated challenge should be addressed. At present, there are two major directions in the search for suitable approaches: (i) ex vivo pretreatments of CAR-T cells to improve their therapeutic functionality when facing such an obstacle as the intratumoral ECM, and (ii) pretreatments aimed at the intratumoral ECM to attenuate its inhibitory effects on CAR-T cells. The following subsections give an overview of both these directions.

### 4.1. Ex Vivo Preconditioning or Engineering T-Lymphocytes for Their Better Infiltration into Solid Tumors

**Figure 5:**
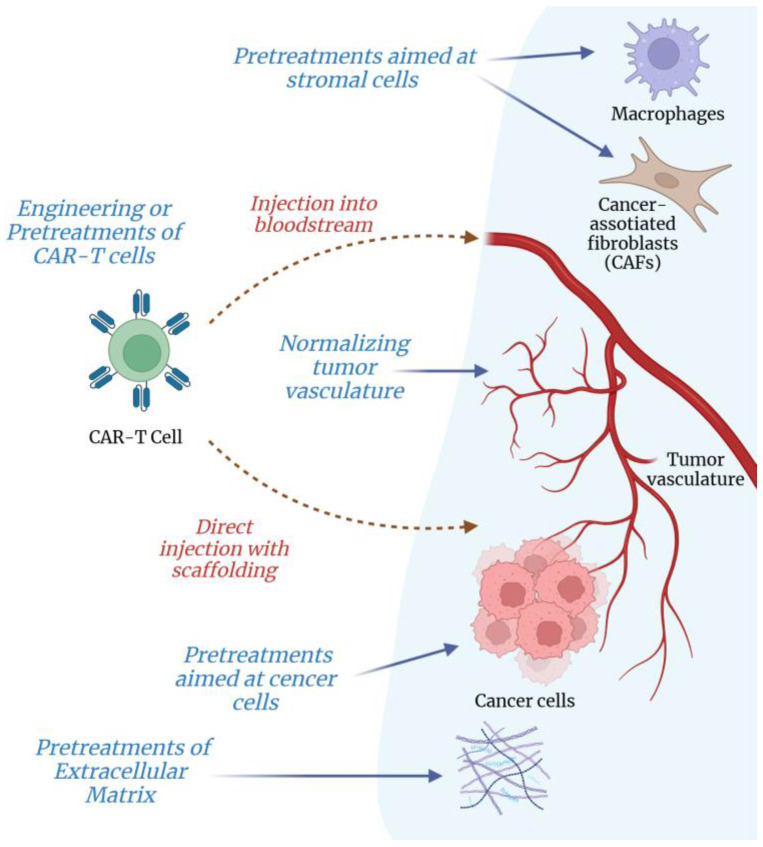
Key strategies proposed as potentially feasible ways to improve efficacy of CAR-T cell therapy against solid tumors. Within each strategy there may be several therapeutic modalities (see accompanying text in Section 4). Created with Biorender.com.

A number of potentially feasible interventions were described to improve the ability of T cells to enter the target tumor and navigate through the intratumoral ECM (Figure 5). These interventions vary in complexity, duration, and tumor-specific applicability.

#### 4.1.1. Preconditioning and Inhibitory Treatments of T-Lymphocytes

Velasquez et al. (2016) reported that the hypoxic preconditioning of natural killer (NK) cells stimulates their migration through the ECM along with an increase in their cytotoxicity toward target cancer cells [86]. It is yet unknown whether an analogous effect can be realized for tumor-directed CAR-T lymphocytes; however, if yes, it would be an attractive method that seems quite feasible in ex vivo systems. The rationale of such hypoxic preconditioning appears to be based on the beneficial pre-stimulation of stress-adaptive responses (HIF-mediated response, HSF1-mediated response, unfolded protein response, and others) in T-cells that render them more tolerant, mobile, and functional under stressful conditions of the TME. Supposedly, the stress-preconditioned T-cells somehow render the intratumoral ECM more passable for them, so that they approach their targets faster.

A rather intriguing publication was made by Zhao et al. (2021) on the effects of a tubulin microtubule inhibitor, vinblastine [84]. The researchers found that the destruction of microtubules in vinblastine-treated cytotoxic T-lymphocytes provides them with a better capability of migrating through dense 3D collagen matrix along with more effective killing of cancer cells in an in vitro model. Mechanistically, it is not clear how the drug-induced collapse of the microtubular network in T-lymphocytes can improve their traffic through 3D collagen. However, if it does work, pharmacological pretreatment with vinblastine may be adopted for CAR-T cells as a very simple ex vivo manipulation, enabling immunotherapists to successfully target ECM-enriched solid tumors.

It follows from a recent publication [80] that ECM stiffness-associated biomechanical stress and the stress-responsive involvement of Osr2 in HDAC3-mediated alterations in the epigenetic regulation of CAR-T cells promote and aggravate their exhaustion within solid tumors. If so, one can suppose that pretreatments of CAR-T cells with certain inhibitors targeting Osr2 or HDAC3 may at least delay the terminal CAR-T cell exhaustion, thereby increasing the efficacy of CAR-T cell therapy toward solid tumors. However, this supposition needs to be examined in the relevant models.

#### 4.1.2. Use of Artificial Hydrogels

Other research groups described the ex vivo preconditioning of T cells by means of placing them into an artificial matrix prepared from a hyaluronic acid (HA)-based hydrogel [87], or into an artificial matrix hydrogel conjugated with a peptide-loaded MHC complex that mimics the ECM of lymph nodes [88]. In the latter case, the additional pre-stimulation of T cells was used with anti-CD28 and tethered interleukin 2 (IL2) [88]. It was asserted in both publications that such ex vivo contacts of T-lymphocytes with the artificially engineered matrix trigger specific signaling in the cells and influence their phenotype/activity; as a result, this stimulates and enhances the subsequent expansion of CD8+ T cells into target tumors.

The HA-based hydrogel containing CAR-T cells was also used for implantation into the tumor cavity: those HA hydrogel implants gradually released CAR-T cells targeting chondroitin sulfate proteoglycan 4 and this approach enabled to prevent the post-surgery tumor recurrence in a murine model [89]. Actually, injectable hydrogels are suggested as convenient carriers for T cells to be employed in anticancer immunotherapy [90]. As an alternative way to the generally accepted introduction of CAR-T cells into the bloodstream, the direct injections of CAR-T cell-containing hydrogel implants into target tumors may help to bypass the problem of CAR-T cell penetration through the subendothelial basement membrane and adjacent ECM. It seems likely that either traditional introducing CAR-T cells into the bloodstream or direct injection/implantation of them into a target tumor will be suitable for different types of solid malignancies; however, this idea needs confirmation in the relevant trials.

#### 4.1.3. Gene Engineering Tumor-Directed CAR-T Cells for Their Better Infiltration into Solid Tumors

Various gene-engineering strategies were suggested to obtain the pools of cytotoxic T-lymphocytes with the acquired ability to better penetrate and persist in the dense and immunosuppressive ECM of solid tumors [11,43,60,91,92,93]. There are at least three main trends among the suggested gene-engineering strategies for CAR-T cells. The one promising approach is to genetically engineer CAR-T cells for expressing and secreting certain enzymes that can degrade ECM components, such as heparanase or hyaluronidase, or matrix metalloproteinases (MMPs), to make a path through the dense stroma. The second trend is when CAR-T cells are genetically engineered to secrete certain cytokines and immunomodulators that can beneficially remodel the tumor microenvironment or enhance their motility and infiltration capabilities toward the intratumoral ECM. Finally, the third trend focuses on gene-engineering CAR-T cells with novel, specifically oriented surface receptors that can recognize and bind components of the intratumoral ECM including cell adhesion factors and chemokines that would facilitate T-cells navigation and retention within the solid tumor.

Some of the suggested gene-engineering approaches were already tested in experimental models and demonstrated encouraging results [11,43,93]. Back in 2015, heparanase expressed by CAR-T lymphocytes was shown to improve their capability of tumor infiltration as well as their antitumor activity toward human neuroblastoma xenografts in mice [68]. The achieved beneficial effects were associated with the newly acquired capability of heparanase-expressing CAR-T cells to split heparan sulfate proteoglycans to pave their way through the ECM-rich tumor stroma [68]. Later, Zhao et al. constructed mesothelin-specific CAR-T cells overexpressing a secreted form of human hyaluronidase PH20; thanks to the conferred hyaluronidase overexpression/secretion, those anti-mesothelin CAR-T cells exhibited better infiltration into tumors and repressed tumor growth in mice with gastric cancer xenografts [94]. Taking into consideration that neither heparanase-expressing CAR-T cells nor hyaluronidase-expressing CAR-T cells are exploited so far, new CAR-T cells expressing some other (supposedly more suitable) ECM-degrading enzymes should be tested for their applicability in antitumor CAR-T cell therapy.

Recently, Zheng et al. reported engineering CAR-T cells that express synthetic Notch (synNotch) receptors inducing the tumor-specific secretion of ECM-degrading enzymes at the tumor locus [95]. Such synNotch CAR-T cells are able to effectively eliminate solid tumors, as the synNotch-mediated enzyme secretion results in the degradation of intratumoral ECM. Moreover, synNotch receptor expression was shown to significantly increase CAR-T cell infiltration that was accompanied by tumor regression without toxic effects in vivo [93]. In principle, such a strategy seems fairly hopeful, though, there is some risk that the artificially imposed expression and secretion of ECM-degrading enzymes will lead to the cleavage of functionally important receptors and glycocalyx on the CAR-T cell surface.

Concerning the engineered secretion of immunomodulators, Chen et al. published the creation of CD19-directed CAR-T cells secreting a bispecific protein of anti-PD1 fused to TGFβ trap [62]. Such a bifunctional secretory product simultaneously co-targets both PD1/PD-L1 pathway and TGFβ-stimulated pathways, thus allowing those CAR-T cells to largely withstand TME-associated immunosuppression [62,92]. It was found in a murine xenograft model of prostate cancer that the secretion of anti-PD1/TGFβ trap-fusion protein by CD19 CAR-T cells strengthened their expansion and infiltration into the tumor as well as their antitumor activity in vivo [96]. Later, the third generation CAR-T cells targeting interleukin 13 receptor alpha 2 (IL13Rα2) and with a CD28 transmembrane domain were shown to be effective against glioblastoma in mouse xenograft models [97]. Importantly, those CAR-T cells exhibited the elevated expression of interferon-gamma (IFN-γ) and improved migration capacity through the stromal ECM that were associated with the achieved positive effect toward glioblastoma xenografts [97].

What about engineering CAR-T cells that are directed to cell adhesion factors or ECM components in solid tumors? Martin-Otal et al. created CAR-T cells targeting extra domain A (EDA) of fibronectin; this domain is a splice variant being expressed in many malignancies and also in the endothelial basement membrane of tumor vasculature [81]. Such EDA-recognizing CAR-T cells had an improved migratory capacity to the site of EDA expression and demonstrated good results in models with murine teratocarcinoma and two human hepatocarcinoma cell lines: both the enhanced cancer cell killing in vitro and the tumor growth-repressing effects in tumor-bearing immunocompetent mice were observed. In vivo, the EDA-CAR-T cells possessed high selectivity in their cytotoxicity due to a tropism for EDA-expressing tumors. Additional benefits from the action of EDA-CAR-T cells were manifested in the inhibition of tumor-stimulated angiogenesis and reduced gene signatures related to the EMT, collagen synthesis, and ECM organization [81]. Another variant of CAR-T cells directed to ECM components were the above CAR-T cells targeting chondroitin sulfate proteoglycan 4; those CAR-T cells performed fairly well when being released from the HA hydrogel implants in a murine model of post-surgery tumor recurrence [89]. In a recent publication by Wickman et al. [98], the engineered CAR-T cells recognized the oncofetal form of tenascin C, a protein component of the ECM, whose C-domain (C.TNC) is expressed in pediatric sarcomas and brain tumors. In vivo, those C.TNC-CAR-T cells exerted improved therapeutic effects against the C.TNC-positive tumors [98].

Thus, there is a variety among the ex vivo manipulations and molecular targets/tools in approaches to overcoming the ECM-created barriers for antitumor CAR-T therapy. The suitable (clinically feasible) variants or combinations remain to be established.

### 4.2. Treatments of Intratumoral ECM to Improve T-Lymphocyte Infiltration and Killing of Cancer Cells

As the tumor-associated ECM is known to impede the penetration and cancer cell-killing function of CAR-T cells, certain strategies are suggested and developed to overcome this challenge. The rational strategies herein are thought to be (i) attenuating the immunosuppressive effects of the intratumoral ECM, (ii) softening the dense ECM/fibrosis in tumors, and (iii) normalizing the aberrant tumor vasculature. However, there are many different approaches to the practical implementation of each and these approaches can also be divided into at least three conditional groups.

#### 4.2.1. Pretreatments of Intratumoral ECM

It seems logical to involve ECM-degrading enzymes to make the intratumoral ECM softer and less “hostile” for entering CAR-T cells. An alternative to the use of such enzymes expressed and secreted by CAR-T cells or certain stromal cells within target tumors may be intratumoral, introducing either exogenous enzyme. The direct intratumor injections of bacterial collagenase were proposed as a remedy for degrading fibrillar collagen to facilitate penetrating an anticancer agent [99]. However, that approach has not been further developed in terms of CAR-T therapy, probably through the extremely high immunogenicity of bacteria-derived collagenases. Later, the direct injections of nattokinase into murine 4T1 tumors (an in vivo model of breast cancer in mice) led to the degradation of fibronectin and attenuation of the tumor stiffness [100]. In the case of intratumoral injections of nattokinase into MDA-MB-231 tumor xenografts (a model of human triple negative breast cancer), boosted CAR-T cell infiltration was observed in the tumor zones along with tumor growth inhibition [100]. Taken together, those findings characterize the intratumoral injections of nattokinase as one of the promising approaches that may help to adopt CAR-T cell therapy for solid tumors.

But how to introduce an exogenous enzyme into a target tumor without syringe needle intervention? Yang et al. published the design/use of a programmed nanoremodeler (DAS@P/H/pp) that is triggered by the acidic intratumor environment, releasing hyaluronidase in response to low pH-induced charge reversal [101]. In murine models of pancreatic cancer, the device-released hyaluronidase strenuously degraded the intratumoral ECM that allowed immunostimulatory molecules to deeper penetrate into the treated tumors. In the same models, the device-evoked degradation of the intratumoral ECM boosted both the recruitment of natural killer (NK) cells and their cancer cell-killing activity [101]. One can wonder whether this hyaluronidase-releasing nanoremodeller would also be able to boost the infiltration and functionality of tumor-directed CAR-T cells? Apparently, thorough examinations are required to clarify the question.

As for non-enzymatic pretreatments of the intratumoral ECM, an original approach was recently described by Li et al. [102] who used a MAtrix REgulating MOtif (MAREMO)-mimicking peptide, which binds to the ECM protein tenascin-C and by so neutralizes pro-tumor activities of the latter via inhibiting interactions of tenascin-C with fibronectin, TGFβ, CXCL12, and others. Among the antitumor effects of this peptide, there was the attenuation of intratumoral ECM-associated immunosuppression and an increase in tumor-infiltrating CD8+ T cells [102]. Such beneficial modulation of the intratumoral ECM by MAREMO-mimicking peptides may improve the outcome of CAR-T cell therapy toward solid (tenascin C-enriched) tumors, although this suggestion needs to be examined in relevant models.

#### 4.2.2. Targeting Cancer and Stromal Cells to Attenuate ECM Synthesis and Fibrosis in Tumor

While focal adhesion kinase (FAK) hyperactivation in human pancreatic ductal adenocarcinoma (PDAC) was found to be associated with the formation of immunosuppressive, fibrotic TME and poor CD8(+) cytotoxic T cell infiltration, the use of the selective FAK inhibitor VS-4718 reduced tumor fibrosis and decreased the amounts of tumor-infiltrating immunosuppressive cells that were accompanied by limited tumor progression and doubling of survival in a mouse model with human PDAC xenografts [52]. Therefore, FAK inhibition may enhance immune surveillance by overcoming fibrotic and immunosuppressive TME in solid tumors that will render them more responsive to CAR-T cell therapy.

According to the recently described mechanism of 3D collagen-triggered CAR-T cell exhaustion [60], some inhibitors of the production of KynA or AHR pathway may increase the efficacy of CAR-T cell therapy toward gastric cancer. 

It is possible to soften the intratumoral ECM by inhibiting the synthesis or assembly of its constituent(s). In a murine xenograft model of PDAC, 4-methylumbelliferone, a selective blocker of hyaluronan synthesis, was found to decrease the intratumoral amounts of hyaluronan and boosted the intratumor infiltration of inoculated γδ T cells that were correlated to the observed suppression of PDAC growth [103]. Those findings suggest that small molecule inhibitors of ECM synthesis can sometimes be used as an auxiliary pharmacological pretreatment aimed at sensitizing solid tumors to CAR-T cell therapy.

Notably, macrophages are known to actively secrete matrix metalloproteinases (MMPs) degrading the ECM [104]. Therefore, these cells may be used as a tool for the MMP-mediated destruction of the intratumoral ECM, if you send them in to target malignancies. Such macrophages were really engineered: they have a chimeric antigen receptor (CAR) which, being activated by its interaction with the tumor antigen HER2, triggers the internal signaling of CD147 and ensures the enhanced expression of MMPs [105]. In a murine model with HER2-4T1 breast cancer, those CAR-147 macrophages were shown to reduce the intratumor collagen depositions, while increasing the intratumor T cell infiltration that was accompanied by the inhibition of in vivo malignant growth [105]. If such ECM-destructing CAR-macrophages can similarly act as allies of CAR-T lymphocytes, this would advance CAR-T therapy of solid tumors.

Among the suggested approaches to softening the intratumor ECM, there is targeting cancer-associated fibroblasts (CAFs) that are the main ECM producers in the TME. For example, Hu et al. reported engineering the cell membrane-anchored and tumor-targeted interleukin 12 (IL12) T cells, which interact with cell-surface vimentin (CSV) expressed by tumor cells in a xenograft model of osteosarcoma [106]. It was demonstrated that the treatment with those CSV-binding IL12 T cells, thanks to their interactions with CSV+ tumor cells, augmented interferon γ (TNFγ) production, while inhibiting transforming growth factor β (TGFβ) secretion and stimulating FAS-mediated apoptosis in abundant CAFs. As a result, the achieved apoptotic elimination of CAFs remodeled the TME to favoring T cell infiltration and repressing osteosarcoma growth [106]. Most likely, such CAF-targeting CAR-T cells would be effective not only for osteosarcoma but for some other solid tumors as well. Under combining or alternating different tools of immunotherapy, the pretreatment with CAF-targeting CAR-T cells may facilitate the subsequent cancer cell-killing action of other CAR-T cells engineered to target malignant cells.

Likewise, pretreatments with the inhibition of fibrosis-promoting processes in ECM-rich tumors may help CAR-T therapy. Indeed, the pretreatment-induced inhibition of the excessive production of collagen and fibronectin by intratumor cells and/or assembly of those ECM proteins into fibers that undergo covalent cross-linking would prevent fibrosis in tumor stroma and, consequently, the formation of dense barriers that would be impassable for CAR-T cells. As fibrosis in tumor stroma is thought to be a cancer-promoting phenomenon, certain therapeutic strategies are developed to combat this. Taking into account that it is TGFβ that is a master regulator of cellular pathways resulting in fibrosis, the synthesis/secretion of TGFβ, TGFβ-receptors, and TGFβ-related signaling seem to be the relevant parameters/targets in the context of fighting the fibrotic tightness of tumor stroma [107]. In some models of CAR-T therapy, the revealed facts of the treatment-induced decrease in intratumor TGFβ production were associated with the achieved ECM destruction and beneficial effects of CAR-T cells [106,108]. Apart from the suggested sensitization of solid tumors, inhibitory targeting TGFβ-mediated pathways may elevate the efficacy of CAR-T cell therapy against certain forms of leukemia if this attenuates TGFβ-provoked fibrosis in bone marrow [82]. Various small molecule inhibitors and antagonists of TGFβ production/receptors/signaling are currently developed and being tested in cancer-related models [107,109]. Perhaps in the future, pretreatments with some of the eventually selected inhibitors will help to avoid intratumor fibrosis, thus enabling tumor-directed CAR-T cells to act more effectively against solid tumors.

Besides TGFβ, other regulators of collagen synthesis/assembly or certain enzymes cross-linking ECM proteins appear to also be potential molecular targets to suppress fibrosis in tumor stroma. For instance, selective inhibitors of HSP47 and/or eHSP90 may beneficially be used to impair tumor fibrosis; the cell-impermeable inhibitors of eHSP90 seem especially promising herein, as such drugs are expected to have low toxicity [47,48,50]. In turn, inhibitors of the ECM-cross-linking enzymes, LOXs [110], and transglutaminases [111] are suggested as potential tools to combat tumor desmoplasia. It seems likely that the pretreatments with such inhibitors of tumor fibrosis may contribute to the improved outcome of CAR-T cell therapy.

#### 4.2.3. Normalizing Tumor Vasculature to Make Its Subendothelial Basement Membrane More Penetrable for T-Lymphocytes and CAR-T Cells

After the endothelial cell monolayer in blood and lymphatic vessels, the subendothelial basement membrane is the first ECM barrier that leukocytes (and CAR-T cells) must overcome to enter the tumor parenchyma. Importantly, crossing the subendothelial basement membrane by T cells may be difficult through the specific peculiarities in the structure/functioning of tumor vasculature [35,37,38,42].

There was an original approach aimed at normalizing the aberrant tumor vasculature whose subendothelial basement membrane often lacks laminin α4, an ECM protein component permissive for T-lymphocyte infiltration [38,64]. It was found in a murine model that the overexpression of the extracellular superoxide dismutase 3 (SOD3) in the endothelial cells of tumor vasculature conferred transcriptional induction of laminin α4; subsequent embedding of laminin α4 into the subendothelial basement membrane facilitated the transmigration of T cells from intratumor vessels into the stroma, thereby boosting T-lymphocyte infiltration of tumors [64]. As for humans, SOD3 expression was correlated to both CD8+ T cell density in tumors of patients with stage II colorectal cancer and their tumor-free survival [64]. If overexpressed SOD3 similarly boosts tumor infiltration by CAR-T cells, this extracellular enzyme may become a very helpful remedy to apply CAR-T therapy against solid malignancies.

#### 4.2.4. Other Potential Agents to Target Tumor-Associated ECM: Oncolytic Viruses, Biotechnological and Nano-Technological Devices … What Else?

In support of an idea that oncolytic virotherapy can successfully be combined with CAR-T cell therapy [112,113], some researchers observed and discussed the beneficial influence of oncolytic viruses on the intratumoral ECM of target tumors. Uche et al. demonstrated that under the combination of herpes simplex virus 1 (HSV-1)-based oncolytic therapy with CAR-T cell therapy, the HSV-1-induced changes in the vasculature and ECM of infected tumors may enhance both the intratumoral infiltration of tumor-reactive CAR-T cells and their antitumor effect [114]. Indeed, if oncolytic viruses cause massive lysis of tumor cells, this appears to be accompanied by the release of intracellular proteases and Ca2+ ions, which in turn destroy the surrounding ECM; such a scenario may facilitate CAR-T cell infiltration/functioning within tumor stroma. In a murine model with renal carcinoma [108], combining oncolytic adenoviral therapy with CAR-T cell therapy resulted in the inhibition of the collagen fiber distribution, thereby altering the ECM structure and increasing CAR-T cell amounts in the target tumor when compared with the effects of the mono-treatment with CAR-T cells. Consequently, the longer survival took place in groups of mice that received the combined treatment [108]. Based on those encouraging data, the combination of CAR-T cell therapy with oncolytic virotherapy can be considered a very promising approach to overcoming the ECM-related challenge of solid tumors.

Intriguingly, biotechnologists are trying to propose their original devices assisting CAR-T lymphocytes to better overcome the ESM barrier in solid malignancies. Wang et al. constructed the so-called in situ Au bioreactor that is able to reprogram ECM viscosity [115]. This intratumorally synthesized device is intended to remodel the stromal ECM in zones of incomplete microwave ablation, thereby allowing CAR-T cells to effectively act in preventing tumor recurrence [115].

As for “nano-technological” ex vivo manipulations, there is a description of nanoengineered CAR-T cell-biohybrids where the T-lymphocytes are connected via biorthogonal conjugation with indocyanine green nanoparticles (INPs) that act as a nano-photosensitizer and a microenvironment modulator [116]. Those biohybrids, after the laser-triggered activation of them, yielded the mild photo-thermal destruction to the stromal ECM and partial normalization of the tumor vasculature without decreasing their cancer cell-killing activity. Such CAR-T-INP biohybrids seem to be promising tools for immunotherapy of solid tumors, thanks to their ability to destroy the intratumoral ECM.

One can see efforts of biotechnologists that are currently focused on the creation of nano-devices being able to beneficially remodel the intratumoral ECM in response to an external stimulus (e.g., laser irradiation) or microenvironmental alterations (e.g., acidosis). The previously mentioned nano-remodeler [101] and nano-photosensitizer [116] are good examples of such approaches aimed at the selective destruction of the intratumoral ECM to augment the efficacy of T-cell-based immunotherapy against solid tumors. It is likely that further collaborative efforts by genetic engineers and biotechnologists will lead to progress in the development of new methods and combinations of treatments that will ultimately help expand the use of CAR-T cell therapy toward solid tumors.

## 5. Conclusions and Perspectives

This review presents a dataset proving that the intratumoral ECM is indeed a serious obstacle for applying CAR-T cell therapy against solid tumors. From the data reviewed, it follows that both the immunosuppressive features of intratumoral ECM and its physical stiffness can impede CAR-T cells to enter the target tumor and migrate within it to kill cancer cells. A conjunction of quite a lot of factors, such as peculiarities of the composition, structure and properties of the TME, abnormality of the tumor vasculature, synthetic/secretory activities of cancer and stromal cells and their phenotypic states, and the levels and spectrum of cytokines present, makes the intratumoral ECM poorly penetrable for CAR-T lymphocytes, thus resulting in failure of CAR-T cell therapy toward solid tumors.

Moreover, the recently published results [60,80] show that the intratumoral ECM is involved in the mechanisms of CAR-T cell exhaustion that can significantly limit the efficacy of CAR-T cell therapy against solid tumors.

Nevertheless, the current situation does not seem hopeless. Quite the contrary, the data considered in Section 4 indicate a number of successful attempts made in cancer-related models where by means of some specific treatments, T cells acquired the ability to overcome the critical ECM barriers. Despite the diversity in the models and approaches used, one can see the two major strategies that can yield success: (i) ex vivo manipulations with cytotoxic T-lymphocytes (including gene-engineering, nano-engineering, usage of artificial matrix, hydrogel implants, biohybrids, etc.) to facilitate tumor-directed T cell migration through the intratumoral ECM, and (ii) pre- or co-treatments of the TME that make the intratumoral ECM less immunosuppressive and more passable for injected CAR-T cells (including the use of certain enzymes or oncolytic viruses, targeting stromal cells that produce or modify the intratumoral ECM, the inhibition of tumor fibrosis development, the application of photo-thermic interventions, etc.).

It seems likely that a combination of some promising approaches adopted from both strategies will make the use of CAR-T cells more effective. The urgent task for researchers is to intensify the relevant studies and trials in order to discover the optimal combination(s) that would improve the outcomes of CAR-T cell therapy against solid tumors. Such a known strategy as normalizing the aberrant tumor vasculature can also contribute to solving the ECM-associated problems of CAR-T cell therapy. The above ECM-related mechanisms of CAR-T cell exhaustion [60,80] seem targetable as well.

Given that each malignancy may be unique in its own way, one should be ready to face the situations where, when under treating solid tumors of one type and localization, absolutely different co-treatments will facilitate CAR-T cell traffic through the intratumoral ECM. Preliminary investigations such as ultrasound probing of a solid tumor and/or studying its biopsy samples may help to assess the density and structure of the intratumoral ECM in order to choose the suitable strategy for overcoming the ECM barriers by CAR-T cells. We do not exclude the possibility of discovering predictive ECM-related biomarkers that would allow oncologists, based on biopsy analysis data, to know in advance whether CAR-T cell therapy is applicable to a particular solid tumor or not. Moreover, preliminary testing of newly generated CAR-T cells in 3D models with ECM-mimicking matrices (similar to what was proposed for T-lymphocytes [83,84,85]) may be useful for predicting the effects of CAR-T cell therapy.

Overall, the authors of this review are optimistic toward the problem. Recent advancements in the field of genome editing, nanomedicine, and biotechnology provide hope for the successful development of clinically applicable ways of overcoming the ECM-associated challenge in antitumor CAR-T cell therapy. By overcoming the ECM-associated challenge, the beneficial potential of CAR-T cell therapy can fully be realized, which will open new avenues for the treatment of solid tumors and improve the outcomes of cancer patients.

## Abbreviations

The following abbreviations are used in this manuscript:

AHR	Aryl hydrocarbon receptor
CAF	Cancer-associated fibroblast
CAR	Chimeric antigen receptor
CSC	Cancer stem cell
ECM	Extracellular matrix
EMT	Epithelial-to-mesenchymal transition
EGF	Epidermal growth factor
FAK	Focal adhesion kinase
FGF	Fibroblast growth factor
GAG	Glycosaminoglycan
HDAC	Histone deacetylase
HIF	Hypoxia-inducible factor
HSP	Heat shock protein
HSF1	Heat shock factor 1
IL	Interleukin
KynA	Kynurenic acid
LOX	Lysyl Oxidase
MMP	Matrix Metalloproteinase
PDGF	Platelet-derived growth factor
PGE2	Prostaglandin E2
TGFβ	Transforming growth factor beta
TME	Tumor microenvironment
TNF	Tumor necrosis factor
VEGF	Vascular endothelial growth factor
